# Psychiatric symptoms in autoimmune encephalitis. A case report

**DOI:** 10.1192/j.eurpsy.2022.1805

**Published:** 2022-09-01

**Authors:** H. Torregrosa Martínez, A. Franco Soler, A. Cerame, P. Coucheiro Limeres, I. Hortal De Pablo

**Affiliations:** 1 Hospital Universitario Príncipe de Asturias, Neurology, Alcala de Henares, Spain; 2 Hospital Universitario José Germain, Psychiatry Department, Leganés, Spain; 3 Hospital Universitario Infanta Cristina, Psychiatry, Parla, Spain

**Keywords:** autoimmune encephalitis, neuroleptics, auditive hallucinations, immunoglobulin

## Abstract

**Introduction:**

Early stages of autoimmune encefalitis (AE) often present cognitive and neuropsychiatric symptoms such as personality change, irritability, axiety, depression, behavioral disorders, hallucinations, disorientation, sleep-wake cycle reversals, …). Thus often these cases are first treated as psychiatric disorders.

**Objectives:**

A literature review throughout a case report presentation.

**Methods:**

We present the case of a 25-year old female with a medical history of iron-deficiency anemia who arrives at the emergency service. She presents the following one week of evolution clinical picture: complex auditive hallucinations, behavioral disturbances, sleep disorder and short term memory impairment. Neurological examination, LP and craneal CT are all normal. CSF analysis has no abnormalities. Thus she entered the psychiatric ward. There she was treated with neuroleptics with no improvement of symptoms presenting a severe psychomotor agitation and language impairment. After neurology interconsultation AE is suspected.

**Results:**

She was performed an EEG (left temporal epileptiform activity), CSF (inflammatory pattern), MRI (bilateral temporal lobe hyperintensity). Suspecting limbic encephalitis the presence of anti-NMDAR antibodies was tested , which turned out to be positive. First she was treated with corticotherapy with mild results. Then she was treated with intravenous immunoglobulin improving significantly.

**Conclusions:**

Anti-NMDAR encephalitis is usually a multistage illness. Early in the course of disease psychiatric manifestations are not rare. Therefore the proper diagnosis and approach of AE may requiere a highly organized assessment, starting with detailed history and physical examination and an appropriate testing to exclude other possible relevant pathologies.

**Disclosure:**

No significant relationships.

